# The Effectiveness of the Problem-Based Learning Teaching Model for Use in Introductory Chinese Undergraduate Medical Courses: A Systematic Review and Meta-Analysis

**DOI:** 10.1371/journal.pone.0120884

**Published:** 2015-03-30

**Authors:** Yanqi Zhang, Liang Zhou, Xiaoyu Liu, Ling Liu, Yazhou Wu, Zengwei Zhao, Dali Yi, Dong Yi

**Affiliations:** Department of Health Statistics, College of Preventive Medicine, Third Military Medical University, Chongqing, China; Iran University of Medical Sciences, IRAN, ISLAMIC REPUBLIC OF

## Abstract

**Background:**

Although the problem-based learning (PBL) emerged in 1969 and was soon widely applied internationally, the rapid development in China only occurred in the last 10 years. This study aims to compare the effect of PBL and lecture-based learning (LBL) on student course examination results for introductory Chinese undergraduate medical courses.

**Methods:**

Randomized and nonrandomized controlled trial studies on PBL use in Chinese undergraduate medical education were retrieved through PubMed, the Excerpta Medica Database (EMBASE), Chinese National Knowledge Infrastructure (CNKI) and VIP China Science and Technology Journal Database (VIP-CSTJ) with publication dates from 1st January 1966 till 31 August 2014. The pass rate, excellence rate and examination scores of course examination were collected. Methodological quality was evaluated based on the modified Jadad scale. The I-square statistic and Chi-square test of heterogeneity were used to assess the statistical heterogeneity. Overall RRs or SMDs with their 95% CIs were calculated in meta-analysis. Meta-regression and subgroup meta-analyses were also performed based on comparators and other confounding factors. Funnel plots and Egger’s tests were performed to assess degrees of publication bias.

**Results:**

The meta-analysis included 31studies and 4,699 subjects. Fourteen studies were of high quality with modified Jadad scores of 4 to 6, and 17 studies were of low quality with scores of 1 to 3. Relative to the LBL model, the PBL model yielded higher course examination pass rates [RR = 1.09, 95%CI (1.03, 1.17)], excellence rates [RR = 1.66, 95%CI (1.33, 2.06)] and examination scores [SMD = 0.82, 95%CI (0.63, 1.01)]. The meta-regression results show that course type was the significant confounding factor that caused heterogeneity in the examination-score meta-analysis (t = 0.410, P<0.001). The examination score SMD in “laboratory course” subgroup [SMD = 2.01, 95% CI: (1.50, 2.52)] was higher than that in “theory course” subgroup [SMD = 0.72, 95% CI: (0.56, 0.89)].

**Conclusions:**

PBL teaching model application in introductory undergraduate medical courses can increase course examination excellence rates and scores in Chinese medical education system. It is more effective when applied to laboratory courses than to theory-based courses.

## Background

The problem-based learning (PBL) teaching model was first developed in 1969, and the approach has since become a popular education model internationally [1,2]. According to World Health Organization data, the PBL teaching model has been used in more than 1,700 medical schools globally, and this number continues to grow [3]. The PBL teaching model was first used in higher medical education settings in China in the 1980s. Due to current shifts in approaches to medical education prevalent in China, this model has been extensively applied as an experimental teaching method in Chinese medical schools. The annual number of published Chinese studies focusing the application of PBL teaching methods has increased exponentially from 14 in 2000 to 474 in 2011.

The PBL teaching model is still controversial [4,5]. Numerous studies have found that in medical education settings, relative to traditional, lecture-based learning (LBL) models, the PBL model presents certain advantages with respect to improving student abilities in inactive learning, two-way communication, clinical thinking, and teamwork [6–9]. A study by Abraham et al. suggested that physiology teaching outcomes could be improved through the use of the PBL teaching model [6]. A study by Mehadizadeh et al. demonstrated that anatomy students that had been instructed via PBL teaching methods not only achieved higher examination scores, but were also highly satisfied with this teaching method [8]. Furthermore, a study by the University of Missouri School of Medicine revealed that overtime, the PBL teaching model may improve the passing rate of the United States Medical Licensing Examination [9]. However, other researchers do not consider the PBL teaching model to be superior to the LBL teaching model with respect to the acquisition of theoretical and fundamental knowledge [10–15].

PBL teaching reforms in China have largely been applied to clinical courses, and these reforms have affected levels of teaching effectiveness in similar ways as they have in other countries [16–18]. However, a systematic, quantitative assessment of the outcomes of PBL teaching model application during learning stages of introductory medical courses has not yet been conducted. For this study, meta-analysis methods were applied to compare the effects of PBL and LBL teaching models on course examination results of introductory undergraduate medical courses in China, thereby providing a scientific basis for evaluating the necessity and feasibility of PBL application in such courses.

## Methods

### Inclusion and exclusion criteria

For this study, we used the following definition of PBL provided by Kinkade [19]: a curriculum of carefully selected activities that test the learner’s critical knowledge acquisition, problem-solving, self-directed learning, and team-participation capacities. Students work in small groups, generate hypotheses about the given case and learning objectives, work outside of class hours to fulfill learning objectives, and then reconvene and solve the problem.

Studies included in this review met the following inclusion criteria: 1) examination of PBL use as a teaching method for five-year undergraduate medical curricula applied in Chinese medical schools; and 2) use of randomized or nonrandomized controlled trials (RCTs), in which experimental groups were instructed using either the PBL teaching model alone or using the PBL teaching model in combination with the traditional LBL teaching model while control groups were instructed strictly based on the LBL teaching model. Courses for which PBL was applied were introductory medical courses in physiology, biochemistry, pharmacological, anatomy, medical statistics, etc. Course examinations were used to assess study populations, and data on examination results were reported.

We excluded studies that did not include a control group; that examined postgraduate or other non-undergraduate courses; that involved non-introductory postgraduate medical courses in internal medicine, surgery, diagnostics and clinical practice; and that did not cite objective course examination data and republished studies.

### Search strategy

To identify relevant studies, we searched for publications using the following databases from the earliest available date through 31 August of 2014: PubMed (1st January 1966), the Excerpta Medica Database (EMBASE) (1st January 1966), the China Knowledge Resource Integrated Database (China National Knowledge Infrastructure, CNKI **http://www.cnki.net/**, 1st January 1979) and the VIP China Science and Technology Journal Database (VIP-CSTJ, **http://oldweb.cqvip.com**, 1st January 1979). The search terms “PBL,” “problem-based learning,” “based on problems,” “active learning,” and “learner centered” were used to identify PBL studies, and these were combined with other key terms such as “medical,” “undergraduate,” “Chinese,” and “China.” We also manually searched through the reference lists of retrieved articles to trace potentially relevant papers.

### Data extraction and quality assessment

Literature screening was independently performed by two reviewers (L.Z. and X.L.) in accordance with the inclusion and exclusion criteria; the data were then extracted and cross-checked. Data extraction in consistencies were resolved through discussion, and secondary calculations found during data extraction were resolved in consultation with a third reviewer (Y.Z.).The extracted data included general study information(the title, author name, publication year and literature resources); basic study characteristics (the number of experimental and control groups, participant characteristics, course name and type, study type, intervention process, literature quality assessment characteristics; etc.)and outcomes (the number of “excellent,” “pass” and “fail” scores, or experimental and control group examination sores).On a 100-point scale, “excellent” denotes a score of≥80 points, “pass” denotes a score of≥60 points and “fail” denotes a score of<60 points.

Methodological quality assessments of the included studies were independently performed by two researchers using the modified Jadad scale [20]. The scale included eight items: randomization, blinding, withdrawals, dropouts, inclusion/exclusion criteria, adverse effects and statistical analysis (Table 1). The total score for each article ranged from 0 to 8 and was computed by summing the score of each item. Low quality studies wielded scores of 0 to 3, and high quality studies achieved scores of 4 to 8.

**Table 1:** The modified Jadad scale.

Eight items	Answer	Score
Was the study described as randomized?	Yes	+1
	No	0
Was the method of randomization appropriate?	Yes	+1
	No	-1
	Not described	0
Was the study described as blinding? ^a^	Yes	+1
	No	0
Was the method of blinding appropriate?	Yes	+1
	No	-1
	Not described	0
Was there a description of withdrawals and dropouts?	Yes	+1
	No	0
Was there a clear description of the inclusion/exclusion criteria?	Yes	+1
	No	0
Was the method used to assess adverse effects described?	Yes	+1
	No	0
Was the methods of statistical analysis described?	Yes	+1
	No	0

a: double-blind got 1 score, single-blind got 0.5 score.

### Statistical methods

The outcome measures of this study were course examination results, which were given two expression forms. The first was a dichotomous outcome (“excellent”, “pass” or “fail” evaluation), and the other was a continuous outcome (i.e., examination scores).

RevMan version 5.3 (Cochrane Collaboration, Copenhagen, Denmark) and the meta-analysis module included in Stata 11.0 (College Station, Texas 77845 USA)were utilized for the meta-analysis. The analytical statistics of relative risk (RR) and standardized mean difference (SMD) at 95% confidence intervals (95% CIs)were used to determine the teaching effectiveness of the PBL model for dichotomous and continuous outcomes, respectively. Before the study results were combined, the I-square statistic and Chi-square test of heterogeneity were used to assess the statistical heterogeneity of the included studies. Values of I^2^>50% or P<0.10 were considered to exhibit significant heterogeneity across studies. The total RR or SMD score at 95% CI was calculated using a random-effects model when heterogeneous results appeared. Otherwise, a fixed-effects model was used.

Meta-regression was used to examine the confounding factors’ effect. Confounding factors included the following: degree major, teaching pattern, course type, PBL group tutor scale, study type and modified Jadad score. We also performed subgroup meta-analyses based on these confounding factors. For the subgroup analysis based on teaching patterns, two subgroups based on whether the PBL teaching model was used independently for the experimental group were used. For one subgroup, the comparator was PBL vs. LBL, and the PBL teaching model was used independently for the experimental group. In the other subgroup, the comparator was PBL+LBL vs. LBL, and both PBL and LBL teaching models were used for the experimental group. We utilized funnel plots and Egger’s tests to assess the degree of publication bias both graphically and statistically. A sensitivity analysis was performed by exchanging the combined model (fixed effects model and random effects model).

## Results

### Literature search results

**Fig 1:**
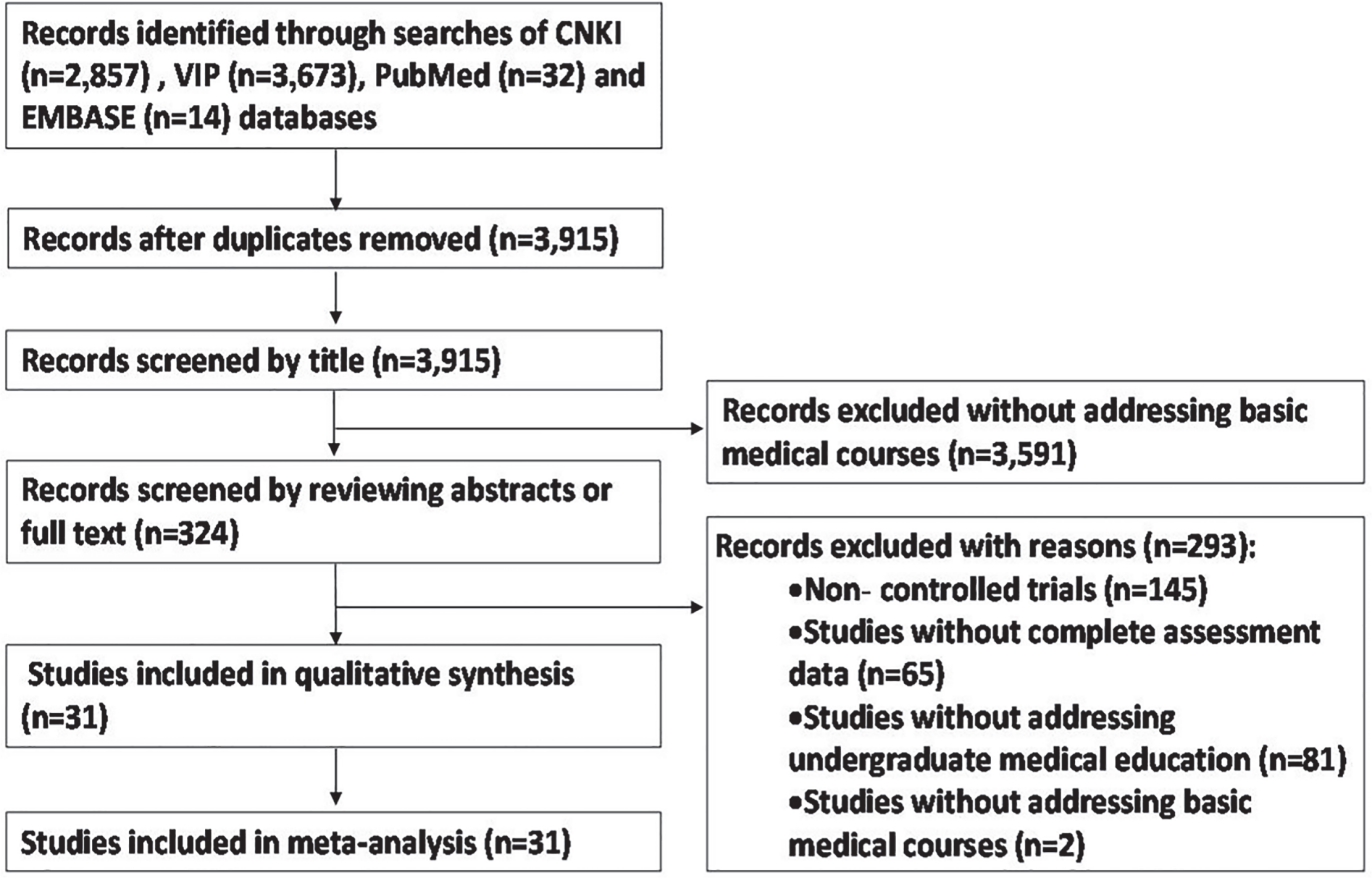
Literature screening process and results.

Using the literature search method, a total of 3,915 relevant studies were initially retrieved. After reviewing the titles, abstracts, and full texts of these studies, 3,884 studies were excluded, and 31 studies were used for the qualitative synthesis and meta-analysis [21–51]. The literature screening process and results are depicted in Fig 1.

### General study characteristics

The general characteristics of the included studies are shown in Table 2. The 31 studies cover 14 disciplines, including clinical medicine, integrative Chinese and western medicine, and pharmaceutical science. Of fifteen courses examined, including those in anatomy, biochemistry, physiology, etc., six are laboratory-based[23,26,31,36,42,50], and 25 are theory-based[21,22,24,25,27–30,32–35,37–41,43–49,51]. Ten of the studies are RCT-based[27,28,31,37,39,43,46,49–51], and the other 21 are non-RCT-based[21–26,29,30,32–36,38,40–42,44,45,47,48].Research subjects included in the studies are freshman to junior-year college students. A total of 4,699 students were included in this meta-analysis, including 2,450 students in the experimental group and 2,249 students in the control group.

**Table 2:** Basic characteristics of the included studies.

ID	Included study	Study time	Study type	Major	Course name	Course type	Number of cases (E/C)	Pattern(E/C)	Tutor scale inPBL group	Grade of students	Course hour in experimental group (PBL/LBL)	Course hour in control group	Outcome	Modified Jadad score
1	Ma 2005[21]	2004	non-RCT	Preventive medicine Pharmacy	Physiology	Theory course	54/53	PBL+LBL/LBL	One tutor in each group	Sophomore	NA/NA	NA	ES	3
2	Zhang 2005[22]	2003	non-RCT	Diagnostic imaging	Pharmacology	Theory course	30/30	PBL+LBL/LBL	One tutor in all groups	NA	6/30	36	ES	3
3	Chen R 2006[23]	2005	non-RCT	Clinical medicine	Pathophysiology	Laboratory Course	35/33	PBL /LBL	One tutor in all groups	Sophomore	5/-	5	PR, ER	3
4	Chen S 2006[24]	2005	non-RCT	Clinical medicine	Physiology	Theory course	174/169	PBL+LBL/LBL	One tutor in all groups	Sophomore	8/NA	NA	ES	2
5	Lv 2006[25]	2005	non-RCT	Stomatology	Medical physics	Theory course	58/56	PBL+LBL/LBL	One tutor in all groups	Freshman	NA/NA	NA	ES	3
6	Cui 2007[26]	2006	non-RCT	Clinical medicine	Biochemistry	Laboratory Course	32/36	PBL /LBL	One tutor in all groups	Sophomore	NA/-	NA	PR, ER	3
7	Lu 2007[27]	2005	RCT	Biomedical engineering	Biochemistry	Theory course	70/35	PBL /LBL	One tutor in each group	Sophomore	NA/-	NA	ES	5
8	Qi 2007[28]	2004–2005	RCT	Clinical medicine	Histology and embryology	Theory course	97/100	PBL /LBL	One tutor in all groups	Freshman	NA/-	NA	ES	4
9	Qin 2007[29]	2005	non-RCT	Clinical medicine	Physiology	Theory course	148/158	PBL+LBL/LBL	One tutor in all groups	Sophomore	NA/NA	NA	PR, ER	3
10	Liu 2008[30]	2006	non-RCT	Anesthesiology, Medical Imaging	Medical advanced mathematics	Theory course	72/68	PBL /LBL	One tutor in all groups	Freshman	NA/-	NA	ES	4
11	Wang 2008[31]	2006	RCT	Clinical medicine	Medical statistics	Laboratory Course	36/36	PBL /LBL	One tutor in all groups	Juniors	NA/-	NA	PR	5
12	Dai 2009[32]	2008	non-RCT	Clinical medicine	Pathology	Theory course	42/40	PBL/LBL	One tutor in all groups	Juniors	NA/-	NA	ES	3
13	Deng 2009[33]	2007	non-RCT	Clinical medicine	Laboratory diagnosis	Theory course	40/40	PBL /LBL	One tutor in all groups	NA	NA/-	NA	PR	3
14	Luo 2009[34]	2006	non-RCT	Medical Laboratory Science	Biochemistry	Theory course	56/58	PBL+LBL/LBL	One tutor in all groups	Sophomore	NA/NA	NA	ES	3
15	Shen 2009[35]	2006	non-RCT	Medical English	Pathophysiology	Theory course	30/29	PBL /LBL	One tutor in all groups	Sophomore	3/-	NA	ES	3
16	Xu 2009[36]	2007	non-RCT	Clinical medicine	Pathology	Laboratory Course	49/49	PBL /LBL	One tutor in all groups	Juniors	NA/-	NA	PR, ER	3
17	Zhou 2009[37]	2007	RCT	Clinical medicine	Anatomy	Theory course	100/100	PBL+LBL/LBL	One tutor in all groups	Freshman	NA/NA	NA	PR, ER	3
18	Zhang 2010[38]	2006–2008	non-RCT	Clinical medicine	Medical statistics	Theory course	39/39	PBL+LBL/LBL	One tutor in all groups	Juniors	8/32	40	PR, ER	4
19	Cui 2011[39]	2010	RCT	Pharmacy	Pharmacology	Theory course	51/48	PBL+LBL/LBL	One tutor in each group	Juniors	NA/NA	NA	ES	3
20	Huang 2011[40]	2011	non-RCT	Chinese medicine	Physiology	Theory course	68/64	PBL+LBL/LBL	One tutor in all groups	Sophomore	NA/NA	NA	ES	4
21	Liu 2011[41]	2010	non-RCT	Integrative Chinese and western medicine	Biochemistry	Theory course	91/70	PBL+LBL/LBL	One tutor in all groups	Sophomore	NA/NA	NA	ES	4
22	Song 2011[42]	2010	non-RCT	Integrative Chinese and western medicine	Pharmacology	Laboratory Course	63/63	PBL /LBL	One tutor in all groups	Juniors	10/-	10	ES	4
23	Tian 2011[43]	2009	RCT	Clinical medicine	Evidence-based medicine	Theory course	46/50	PBL /LBL	One tutor in all groups	Juniors	NA/-	NA	ES	6
24	Wu 2011 [44]	2009	non-RCT	Integrative Chinese and western medicine	Histology	Theory course	100/100/100	PBL/PBL+LBL /LBL	One tutor in all groups	Freshman	NA/-	NA	ES	3
25	Xing 2011[45]	2009	non-RCT	Obstetrics and gynecology	Laboratory diagnosis	Theory course	57/55	PBL+LBL/LBL	One tutor in all groups	Juniors	NA/NA	NA	ES	4
26	Yang 2012[46]	2011	RCT	Clinical medicine	Immunology	Theory course	256/238	PBL+LBL/LBL	One tutor in all groups	Sophomore	NA/NA	NA	PR, ER	5
27	Yan 2013[47]	2011	non-RCT	Orthopsychiatry	Pathology	Theory course	57/53	PBL+LBL/LBL	One tutor in all groups	Juniors	NA /NA	NA	ES	3
28	He 2014[48]	2012	non-RCT	Clinical medicine	Biochemistry	Theory course	92/91	PBL+LBL/LBL	One tutor in all groups	Sophomore	NA/NA	NA	PR, ER	3
29	Qiu 2014[49]	2012	RCT	Clinical medicine	Human developmental genetics	Theory course	124/126	PBL/LBL	One tutor in all groups	Sophomore	NA/-	NA	ES	5
30	Yin 2014[50]	2012	RCT	Rehabilitation medicine	Anatomy	Laboratory Course	32/32	PBL/LBL	One tutor in all groups	Freshman	NA/-	NA	ES	4
31	Zhao 2014[51]	2009	RCT	Clinical medicine	Pharmacology	Theory course	151/130	PBL+LBL/LBL	One tutor in all groups	Sophomore	NA/NA	NA	PR, ER	5

E/C: E mean experimental group, C mean control group

Pattern: teaching pattern, PBL mean PBL teaching model alone in experimental group, PBL+LBL mean PBL+LBL teaching model in combination in experimental group, LBL mean LBL teaching model in control group

Outcome: PR mean pass rate, ER mean excellent rate, ES mean examination score

NA: Not Applicable

Among the 31 studies that were included in the meta-analysis, experimental groups examined in14 of these studies adopted the complete PBL teaching model [23,26–28,30–33,35,36,42,43,49,50], and experimental groups examined in 16 of the studies adopted the mixed PBL+LBL teaching model [21,22,24,25,29,34,37–41,45–48,51]. One of the studies considered two experimental groups [44], with one applying the complete PBL teaching model while the other applied the mixed PBL+LBL teaching model. The control groups used in all 31studies applied the LBL teaching model. In all of the studies, the PBL teaching model was only applied for one semester. While class schedule data were collected, several studies did not provide information on class hours. Outcome measurements were largely collected toward the end of each class. Assessment tools applied were largely tests designed by the researchers themselves.

Eleven studies disclosed the number of "pass" and “fail" grades collected for final course examinations[23,26,29,31,33,36–38,46,48,51], nine of which also reported the number of “excellent” grades collected[23,26,29,36–38,46,48,51]. In total, 23studies disclosed numerical examination scores for final course examinations[21,22,24,25,27,28,30,32,34,35,38–47,49–51]. Of these 23 studies, three reported on the number of “excellent,” "pass" and “fail" grades collected [38,46,51].

### Evaluation of the methodological quality of the included studies

The 31studies examined were evaluated using the modified Jadad scale. From on this assessment, 17studies (54.8%) were assigned scores of 2 or 3[21–26,29,32–37,39,44,47,48], and 14studies (45.2%) were assigned scores of 4, 5 or 6[27,28,30,31,38,40–43,45,46,49–51]. The mean modified Jadad scale score was 3.6, and the standard deviation was 0.9. The modified Jadad scores collected for each study are shown in Table 3.

**Table 3:** Modified Jadad scores of the included studies.

ID	Included study	Was the research described as randomized?^#^	Was the approach of randomization appropriate?*	Was the research described as blinding? ^#^	Was the approach of blinding appropriate? *	Was there a presentation of withdrawals and dropouts? ^#^	Was there a presentation of the inclusion/exclusion criteria? ^#^	Was the approach used to assess adverse effects described? ^#^	Was the approach of statistical analysis described? ^#^	total
1	Ma 2005[21]	0	0	0	0	1	1	0	1	3
2	Zhang 2005[22]	0	0	0	0	1	1	1	0	3
3	Chen R 2006[23]	0	0	0	0	1	1	1	0	3
4	Chen S 2006[24]	0	0	0	0	1	1	0	0	2
5	Lv 2006[25]	0	0	0	0	1	1	1	0	3
6	Cui 2007[26]	0	0	0	0	1	1	1	0	3
7	Lu 2007[27]	1	0	0	0	1	1	1	1	5
8	Qi 2007[28]	1	0	0	0	1	1	0	1	4
9	Qin 2007[29]	0	0	0	0	1	1	0	1	3
10	Liu 2008[30]	0	0	0	0	1	1	1	1	4
11	Wang 2008[31]	1	1	0	0	1	1	1	0	5
12	Dai 2009[32]	0	0	0	0	1	1	1	0	3
13	Deng 2009[33]	0	0	0	0	1	0	1	1	3
14	Luo 2009[34]	0	0	0	0	1	1	1	0	3
15	Shen 2009[35]	0	0	0	0	1	1	1	0	3
16	Xu 2009[36]	0	0	0	0	1	1	1	0	3
17	Zhou 2009[37]	1	0	0	0	1	1	0	0	3
18	Zhang 2010[38]	0	0	0	0	1	1	1	1	4
19	Cui 2011[39]	1	0	0	0	1	1	0	0	3
20	Huang 2011[40]	0	0	0	0	1	1	1	1	4
21	Liu 2011[41]	0	0	0	0	1	1	1	1	4
22	Song 2011[42]	0	0	0	0	1	1	1	1	4
23	Tian 2011[43]	1	1	0	0	1	1	1	1	6
24	Wu 2011 [44]	0	0	0	0	1	1	1	0	3
25	Xing 2011[45]	0	0	0	0	1	1	1	1	4
26	Yang 2012[46]	1	0	0	0	1	1	1	1	5
27	Yan 2013[47]	0	0	0	0	1	1	0	1	3
28	He 2014[48]	0	0	0	0	1	1	1	0	3
29	Qiu 2014[49]	1	0	0	0	1	1	1	1	5
30	Yin 2014[50]	1	0	0	0	1	1	0	1	4
31	Zhao 2014[51]	1	0	0	0	1	1	1	1	5

#: “1” means “Yes”, “0” means “No”;

*: “1” means “Yes”, “0” means “Not described”

### Meta-analysis results

**Fig 2:**
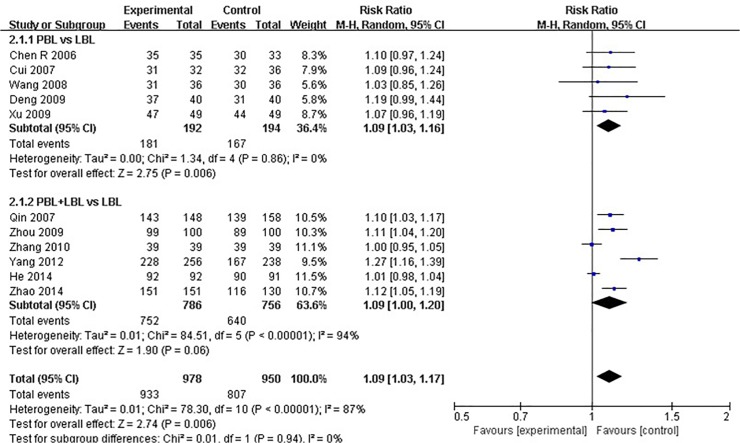
Forest plot of PBL experimental group and LBL control group course pass rates (random effects model). Events: “pass” events, M-H: Mantel-Haenszel, PBL:PBL teaching model independently applied to the experimental group, PBL+LBL: PBL+LBL teaching models applied to the experimental group, LBL: LBL teaching model applied to the control group.

Eleven studies disclosed pass rate data [23,26,29,31,33,36–38,46,48,51]. The average experimental group pass rate was 95.4% [95%CI: (94.1%, 96.7%)], and that of control group was 84.9% [95%CI: (82.6%, 87.2%)] (Table 4). Because a significant degree of heterogeneity was observed across all of the11 studies (I^2^ = 87%, P<0.001),a random effects model was utilized for the meta-analysis. The analytical results reveal that the experimental group produced higher course examination pass rates than the LBL control group [RR: 1.09, 95% CI: (1.03, 1.17)] (Fig 2). For studies that compared PBL and LBL methods, the average PBL group passing rate was 94.3% [95%CI: (91.0%, 97.6%)], and that of the LBL group was 86.1% [95%CI: (81.2%, 91.0%)] (Table 4). No heterogeneity was observed across these studies (I^2^ = 0%, P = 0.860), and the analytical results reveal that the PBL model produces higher course examination passing rates than the traditional teaching model [RR: 1.09, 95% CI: (1.03, 1.16)] (Fig 2). Among studies that conducted PBL+LBL vs. LBL comparisons, the average PBL+LBL group passing rate was 95.7% [95%CI: (94.3%, 97.1%)], and that of the LBL group was 82.7%[95%CI: (82.1%, 87.3%)] (Table 4). Due to the presence of heterogeneity across these studies (I^2^ = 94%, P<0.001), a random effects model was utilized for the meta-analysis. The analytical results reveal that the PBL+LBL model did not produce a significantly higher course examination passing rate than the traditional teaching model [RR: 1.09, 95% CI: (1.00, 1.20)](Fig 2).

**Table 4:** The average pass rate and average excellent rate of experimental group and control group.

PBL pattern	Average Pass rate (95%CI)	Average Excellent rate (95%CI)
PBL vs. LBL		
Experimental group	94.3% (91.0%-97.6%)	59.5% (50.5%-68.3%)
Control group	86.1% (81.2%-91.0%)	30.5%(22.2%-38.8%)
PBL+LBL vs. LBL		
Experimental group	95.7% (94.3%-97.1%)	50.4% (46.9%-53.9%)
Control group	84.7% (82.1%-87.3%)	31.2% (27.9%-34.5%)
Total		
Experimental group	95.4% (94.1%-96.7%)	51.6% (48.3%-54.9%)
Control group	84.9% (82.6%-87.2%)	31.1% (28.0%-34.2%)

**Fig 3:**
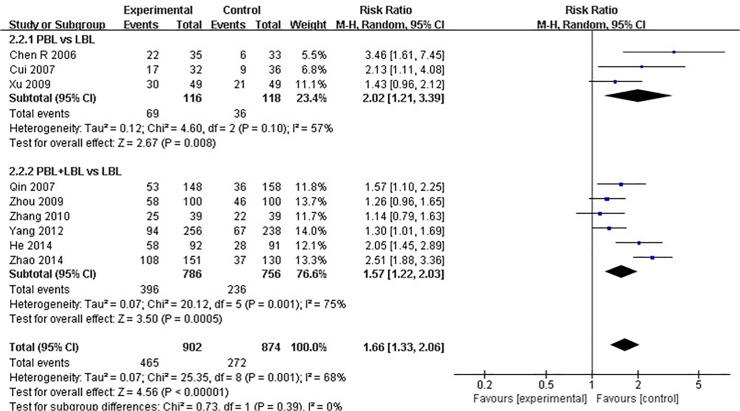
Forest plot of PBL experimental group and LBL control group course excellence rates (random effects model). Events: “excellence” events, M-H: Mantel-Haenszel, PBL: PBL teaching model independently applied to the experimental group, PBL+LBL: PBL+LBL teaching models applied to the experimental group, LBL: LBL teaching model applied to the control group.

Nine of the included studies [23,26,29,36–38,46,48,51] reported course examination excellence rates (≥80 score). The average experimental group pass rate was 51.6% [95%CI: (48.3%, 54.9%)], and that of the control group was 31.1% [95%CI: (28.0%, 34.2%)] (Table 4). A moderate degree of heterogeneity was observed across all nine studies (I^2^ = 68%, P = 0.001). A random effects model was utilized for the meta-analysis, and the analytical results reveal that the experimental group produced a significantly higher course examination excellence rate than the LBL control group [RR: 1.66, 95% CI: (1.33, 2.06)] (Fig 3).Among studies that conducted PBL vs. LBL comparisons, the average PBL group excellence rate was 59.5% [95%CI: (50.5%, 68.3%)], and that of the LBL group was 30.5% [95%CI: (22.2%, 38.8%)] (Table 4). A moderate degree of heterogeneity was detected across these studies (I^2^ = 57%, P = 0.10), and random effects model results reveal that the PBL model generated higher course examination excellence rates than the traditional teaching model [RR: 2.02, 95% CI: (1.21, 3.39)] (Fig 3). Among studies that conducted PBL+LBL vs. LBL comparisons, the average PBL+LBL group excellence rate was 50.4% [95%CI: (46.9%, 53.9%)], and that of the LBL group was 31.2% [95%CI: (27.9%, 34.5%)] (Table 4). A moderate degree of heterogeneity was also observed (I^2^ = 75%, P = 0.001), and the analytical results reveal that the PBL+LBL model produces higher course examination excellence rates than the traditional teaching model [RR: 1.57 95% CI: (1.22, 2.03)] (Fig 3).

**Fig 4:**
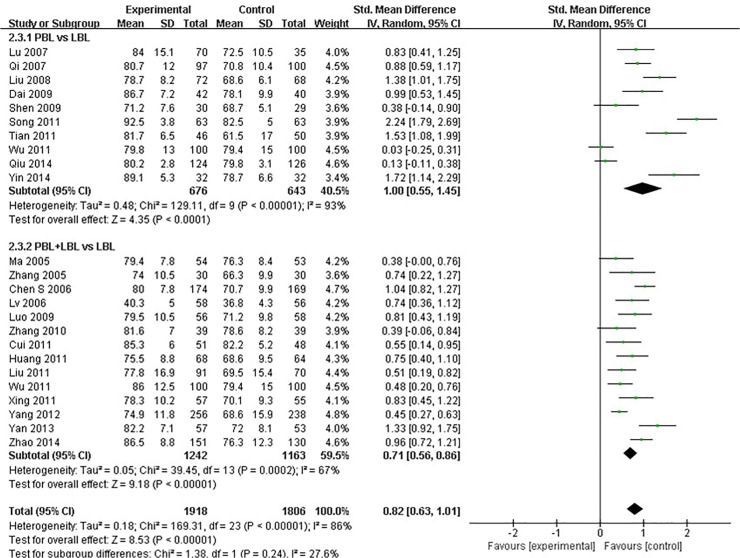
Forest plot of PBL experimental group and LBL control group course examination scores (random effects model). IV: inverse variance, PBL: PBL teaching model independently applied to the experimental group, PBL+LBL: PBL+LBL teaching models applied to the experimental group, LBL: LBL teaching model applied to the control group.

Twenty-three of the studies examined [21,22,24,25,27,28,30,32,34,35,38–47,49–51] reported course examination scores. A high degree of heterogeneity was observed across all of these studies (I^2^ = 86%, P<0.001), and a random effects model was utilized for the meta-analysis. The analytical results reveal that the experimental group produced significantly higher examination scores than the LBL control group [SMD: 0.82, 95% CI: (0.63, 1.01)] (Fig 4).Among studies that conducted PBL vs. LBL comparisons, a high degree of heterogeneity was found (I^2^ = 93%, P<0.001). A random effects model was used for the meta-analysis, and the analytical results reveal that PBL methods produce significantly higher course examination scores than traditional teaching methods [SMD: 1.00, 95% CI: (0.55, 1.45)] (Fig 4). Among studies that conducted PBL+LBL vs. LBL comparisons, a moderate degree of heterogeneity was observed (I^2^ = 67%, P<0.001).Random-effects model results reveal that PBL+LBL methods produce significantly higher course examination scores than traditional teaching methods [SMD: 0.71, 95% CI: (0.56, 0.86)] (Fig 4).

A meta-regression was performed because a relatively high degree of heterogeneity between the included studies was found. The following confounding factors that were considered: degree major, teaching pattern, course type, PBL group tutor scale, study type and modified Jadad score. Among the three outcomes, we discovered that course type is the significant confounding factor that causes examination-score meta-regression heterogeneity (t = 0.410, P<0.001) (Table 5).To further examine heterogeneity from confounding factors, we performed a subgroup meta-analysis based on the following factors: degree major, course type, PBL group tutor scale, study type and modified Jadad score. For most of the subgroups, heterogeneity has not been eliminated. However, for the “laboratory course” subgroup, we did not detect heterogeneity through our meta-analysis of pass rates (I^2^ = 0%, P = 0.950).As well, heterogeneity was not identified through our meta-analysis of “laboratory course” subgroup examination scores (I^2^ = 49%, P = 0.160)or of the substantial heterogeneity group(I^2^ = 18%, P = 0.300). The subgroup examination-score meta-analysis also revealed that the difference in SMD levels between the “theory course”[SMD: 0.72, 95% CI: (0.56, 0.89)] and “laboratory course” subgroups [SMD: 2.01, 95% CI: (1.50, 2.52)]is statistically significance (P<0.001).The examination score SMD value between experimental and control groups for the “laboratory course” subgroup was found to be higher than that of the “theory course” subgroup (Table 6).

**Table 5:** Meta-regression of the effects of confounding factors on pass rate, excellent rate and examination score.

Outcome	Factor	Coefficient (95%CI)	std. error	t	P
**Pass Rate**					
	Major	-	-	-	-
	Pattern	0.014(-0.103–0.132)	0.051	0.280	0.787
	Course type	-0.033(-0.156–0.089)	0.053	-0.620	0.550
	Tutor scale in PBL group		-	-	-
	Study type	-0.069(-0.162–0.025)	0.041	-1.690	0.129
	Modified Jadad score	0.037(-0.015–0.09)	0.023	1.630	0.142
**Excellent Rate**					
	Major	-	-	-	-
	Pattern	-0.231(-0.865–0.403)	0.268	-0.860	0.417
	Course type	0.231(-0.403–0.865)	0.268	0.860	0.417
	Tutor scale in PBL group		-	-	-
	Study type	0.069(-0.508–0.647)	0.244	0.280	0.785
	Modified Jadad score	-0.005(-0.331–0.322)	0.138	-0.030	0.974
**Examination Score**					
	Major	0.042(-0.203–0.288)	0.119	0.360	0.724
	Pattern	-0.272(-0.706–0.163)	0.210	-1.300	0.209
	Course type	1.291(0.637–1.944)	0.315	4.100	0.000
	Tutor scale in PBL group	0.273(-0.391–0.937)	0.320	0.850	0.402
	Study type	-0.04(-0.508–0.429)	0.226	-0.180	0.862
	Modified Jadad score	0.081(-0.154–0.316)	0.113	0.710	0.483

**Table 6:** Subgroup meta-analyses based on confounding factors.

	Pass Rate								Excellent Rate								Examination Score						
	Number of studies	Sample size(T/C)	Number of events (T/C)	Weight(%)	I^2^(%)	P of heterogeneity	RR(95% CI)	P of effect	Number of studies	Sample size (T/C)	Number of events (T/C)	Weight(%)	I^2^(%)	P of heterogeneity	RR(95% CI)	P of effect	Number of studies	Sample size(T/C)	Weight(%)	I^2^(%)	P of heterogeneity	SMD(95% CI)	P of effect
**Major**																							
**Clinical medicine**	11	978/950	933/807	100	87	<0.001	1.09 (1.03–1.17)	0.006	9	902/874	465/272	100	68	0.001	1.66 (1.33–2.06)	<0.001	8	929/892	34.8	88	<0.001	0.78 (0.49–1.07)	<0.001
**Integrative Chinese and western medicine**	0	-	-	-	-	-	-		-	-	-	-	-		-	-	4	354/333	17.3	96	<0.001	0.80 (0.03–1.56)	0.040
**Others**	0	-	-	-	-	-	-		-	-	-	-	-		-	-	12	635/581	47.9	68	<0.001	0.86 (0.65–1.07)	<0.001
**Pattern**																							
**PBL vs LBL**	5	192/194	181/167	36.4	0	0.860	1.09 (1.03–1.16)	0.006	3	116/118	69/36	23.4	57	0.100	2.02 (1.21–3.39)	0.008	10	676/643	40.5	93	<0.001	1.00 (0.55–1.45)	<0.001
**PBL+LBL vs LBL**	6	786/756	752/640	63.6	94	<0.001	1.09 (1.00–1.20)	0.060	6	786/756	396/236	76.6	75	0.001	1.57 (1.22–2.03)	<0.001	14	1242/1163	59.5	67	<0.001	0.71 (0.56–0.86)	<0.001
**Course type**																							
**Theory course**	7	826/796	789/671	69.4	93	<0.001	1.10 (1.01–1.21	0.030	6	786/756	396/236	76.6	75	0.001	1.57(1.22–2.03)	<0.001	22	1823/1711	92.7	81	<0.001	0.72 (0.56–0.89)	<0.001
**Laboratory course**	4	152/154	144/136	30.6	0	0.950	1.08 (1.01–1.15)	0.020	3	116/118	69/36	23.4	57	0.100	2.02 (1.21–3.39)	0.008	2	95/95	7.3	49	0.160	2.01 (1.50–2.52)*	<0.001
**Tutor scale in PBL group**																							
**One tutor in each group**	0	-	-	-	-		-		0	-	-	-	-		-		3	175/136	12.2	18	0.300	0.57 (0.32–0.83)	<0.001
**One tutor in all groups**	11	978/950	933/807	100	87	<0.001	1.09 (1.03–1.17)	0.006	9	902/874	465/272	100	68	0.001	1.66 (1.33–2.06)	<0.001	21	1743/1670	87.8	88	<0.001	0.85 (0.65–1.06)	<0.001
**Study Type**																							
**RCT**	4	543/504	509/402	36	66	0.030	1.14 (1.06–1.24)	<0.001	3	507/468	260/150	41	86	<0.001	1.60 (1.04–2.45)	0.030	8	827/759	33.9	88	<0.001	0.84 (0.52–1.16)	<0.001
**Non-RCT**	7	435/446	424/405	64	75	<0.001	1.06 (1.00–1.12)	0.050	6	395/406	205/122	59	53	0.060	1.69 (1.30–2.19)	<0.001	16	1091/1047	66.1	86	<0.001	0.81 (0.56–1.05)	<0.001
**Modified Jadad score**																							
**<4**	7	496/507	484/455	63.2	74	<0.001	1.08 (1.02–1.15)	0.010	6	456/467	238/146	61	51	<0.001	1.69 (1.33–2.15)	<0.001	11	752/736	45.4	80	<0.001	0.67 (0.43–0.92)	<0.001
**≥4**	4	482/443	449/352	36.8	93	<0.001	1.10 (0.95–1.29)	0.210	3	446/407	227/126	39	87	<0.001	1.56 (0.96–2.52)	0.070	13	1166/1070	5406	90	<0.001	0.94 (0.66–1.23)	<0.001
**Total**	**11**	**978/950**	**933/807**	**100**	**87**	**<0.001**	**1.09 (1.03–1.17)**	**0.006**	**9**	**902/874**	**465/272**	**100**	**68**	**0.001**	**1.66 (1.33–2.06)**	**<0.001**	**24**	**1918/1806**	**100**	**86**	**<0.001**	**0.82 (0.63–1.01)**	**<0.001**

*: The Examination Score SMD of “Laboratory course” subgroup is higher than “theory course” subgroup, P<0.001.

### Publication bias

**Fig 5:**
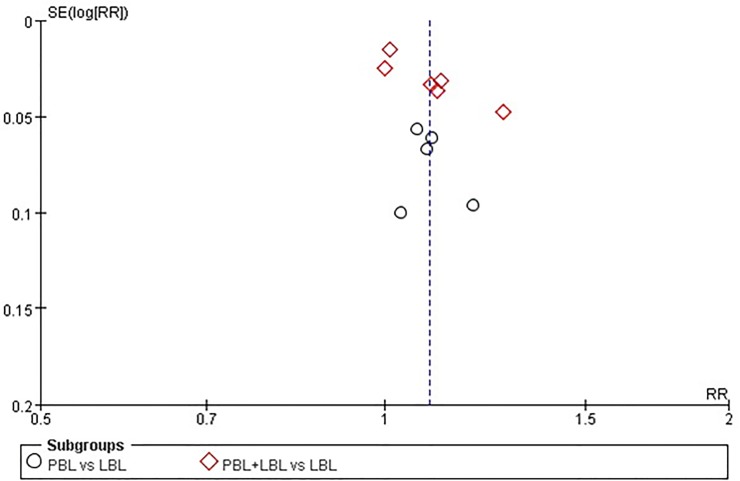
Funnel plot of the meta-analysis of PBL experimental group and LBL control group course pass rates.

**Fig 6:**
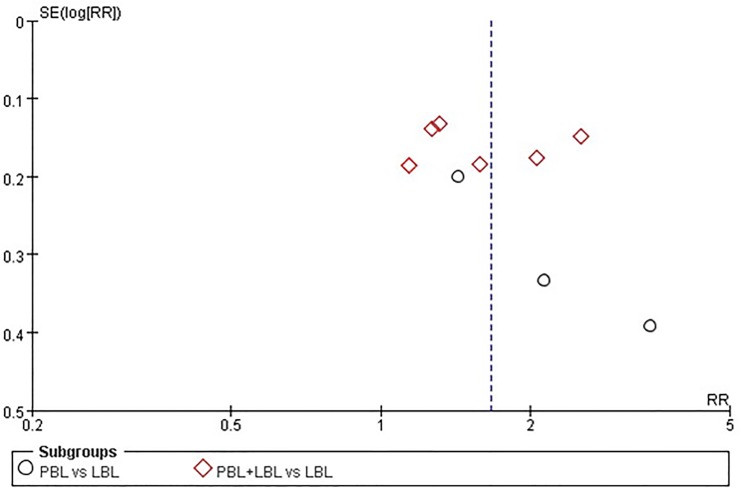
Funnel plot of the meta-analysis of PBL experimental group and LBL control group course excellence rates.

**Fig 7:**
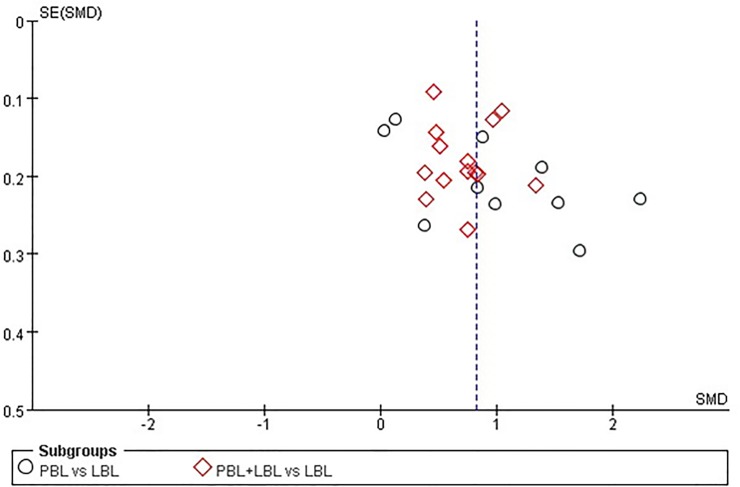
Funnel plot of the meta-analysis of PBL experimental group and LBL control group course examination score.

The pass rate, excellence rate and examination score funnel plots do not reveal a significant degree of publication bias between the included studies (Figs 5–7). However, publication bias Egger’s test results reveal a minor degree of publication bias among pass rate (t = 2.310, P = 0.050) and examination score (t = 2.130, P = 0.045) results (Table 7).

**Table 7:** Egger’s test of pass rate, excellent rate and examination score for publication bias.

Outcome	Number of studies	coefficient of bias (95%CI)	std. error	t	P
**Pass Rate**	11	2.123(0.004–4.243)	0.919	2.310	0.050
**Excellent Rate**	9	2.307(-2.406–7.019)	1.993	1.160	0.285
**Examination Score**	24	3.700(0.093–7.307)	1.739	2.130	0.045

### Sensitivity analysis

**Fig 8:**
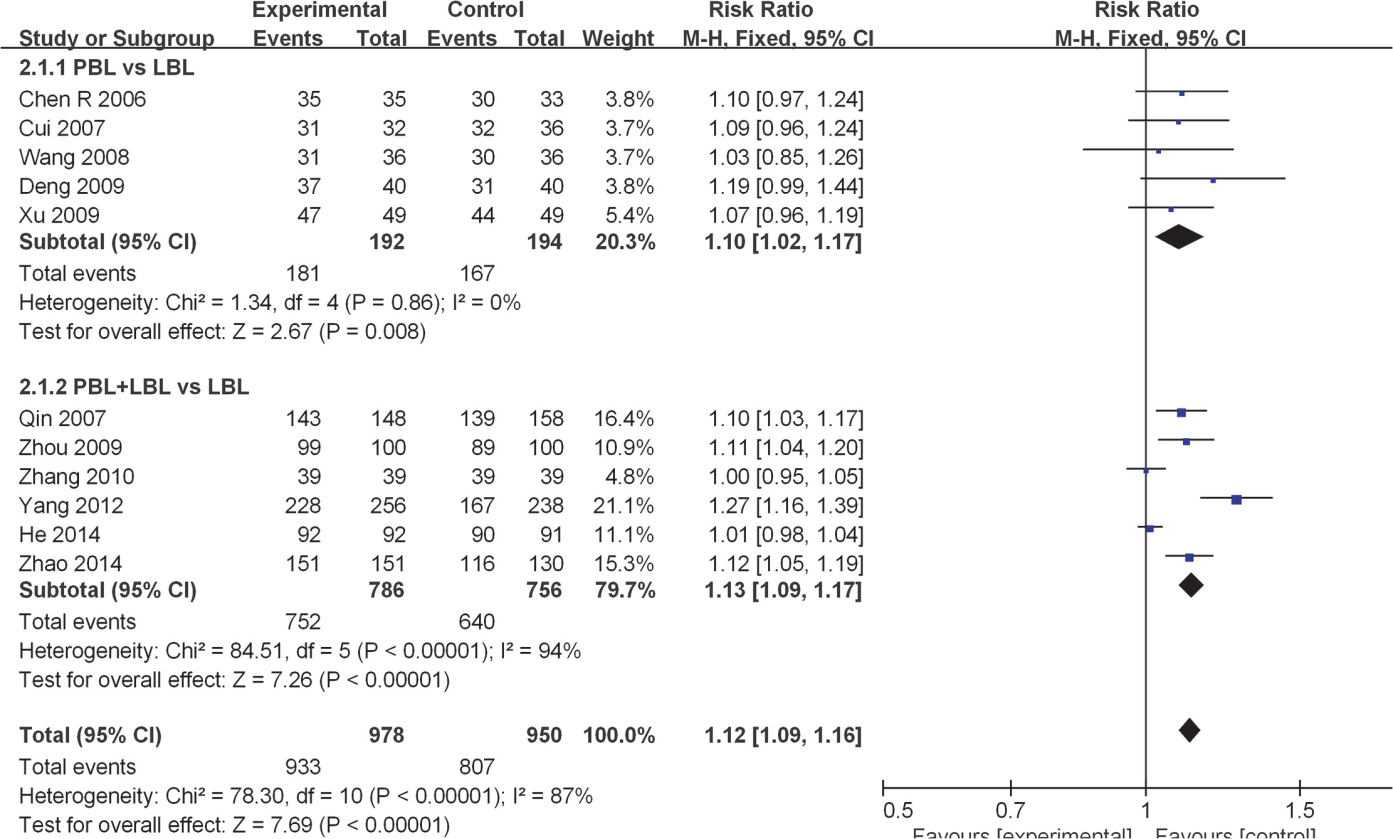
Forest plot of PBL experimental group and LBL control group course excellence rates (fixed effects model). Events: “excellence” events, M-H: Mantel-Haenszel, PBL: PBL teaching model independently applied to the experimental group, PBL+LBL: PBL+LBL teaching models applied to the experimental group, LBL: LBL teaching model applied to the control group.

**Fig 9:**
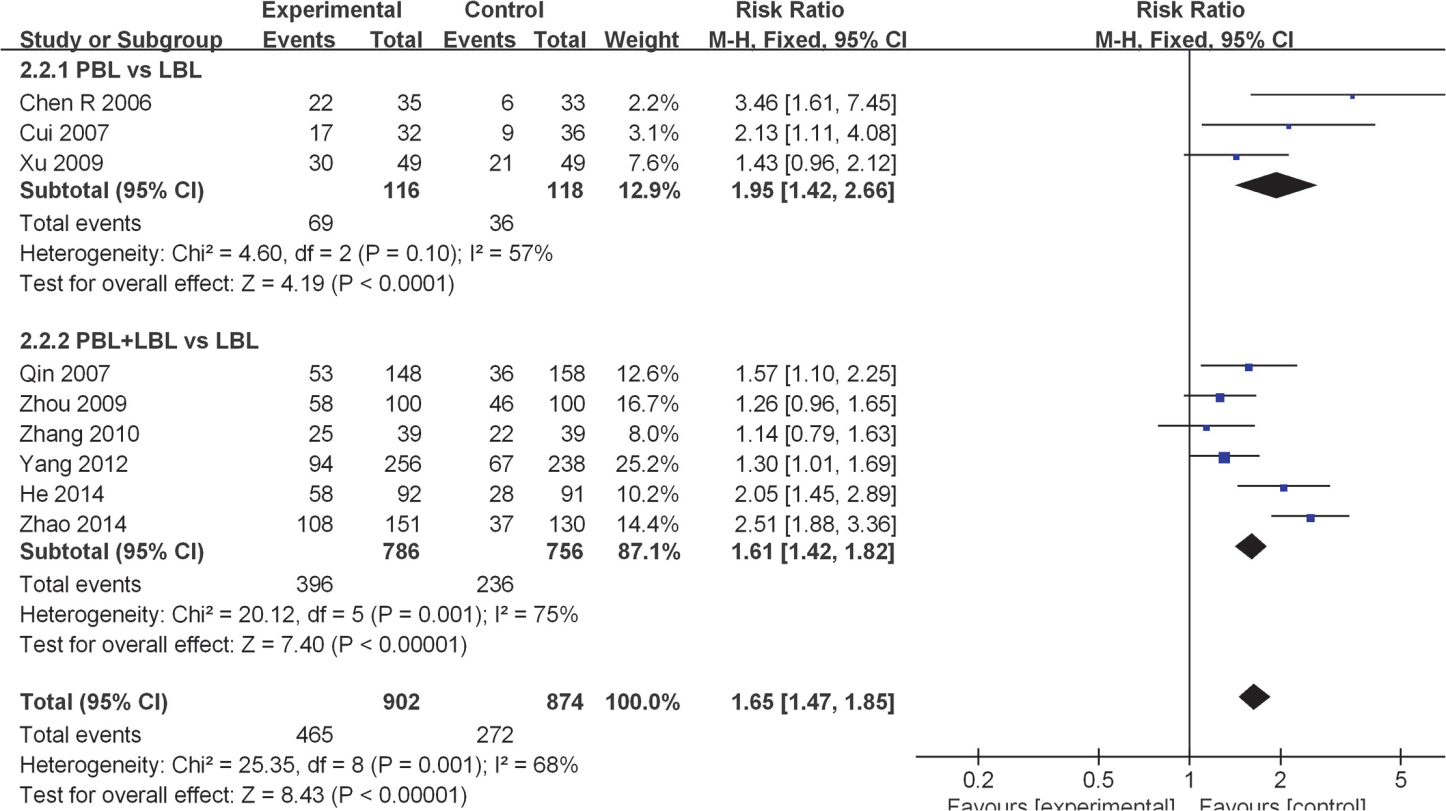
Forest plot of PBL experimental group and LBL control group course excellence rates (fixed effects model). Events: “excellence” events, M-H: Mantel-Haenszel, PBL: PBL teaching model independently applied to the experimental group, PBL+LBL: PBL+LBL teaching models applied to the experimental group, LBL: LBL teaching model applied to the control group.

**Fig 10:**
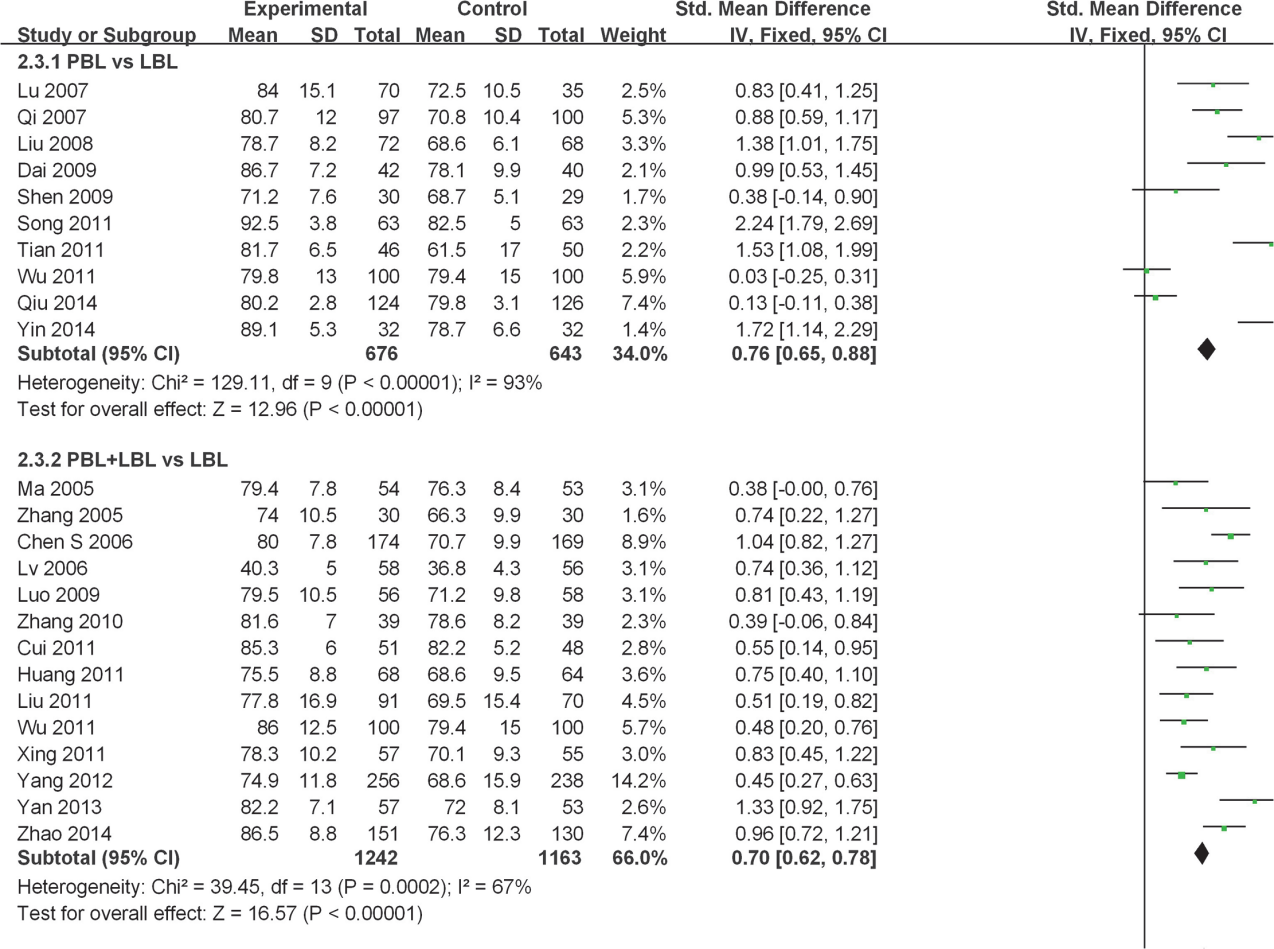
Forest plot of PBL experimental group and LBL control group course examination scores (fixed effects model). IV: inverse variance, PBL: PBL teaching model independently applied to the experimental group, PBL+LBL: PBL+LBL teaching models applied to the experimental group, LBL: LBL teaching model applied to the control group.

A sensitivity analysis was performed by changing the combined model from a random effects model to a fixed effects model. The results of the fixed effects model were consistent with those of the random effects model (Figs 8–10).

## Discussion

Since its first application in Canada in the late 1960s, PBL has been widely adopted innumerous universities internationally. As in China, education systems in various regions and countries differ considerably from those of the U.S. Furthermore, cultural differences influence the effectiveness of PBL methods [52]. Therefore, the target population must be limited to Chinese students of the Chinese education system in order to examine the potential effectiveness of the PBL teaching model in China. Our research method will serve as a valuable reference for researchers based in countries of differing cultural backgrounds who wish to evaluate the effectiveness of the PBL teaching model. Our research conclusions will promote PBL teaching model application in countries of differing cultural characteristics.

The results of our meta-analysis indicate that the PBL teaching model can yield significantly positive results relative to the LBL teaching model, particularly in excellence rates and examination scores, which is inconsistent with the previous finding that PBL students either perform no differently or slightly worse than students in conventional on measures of knowledge such as basic sciences examinations [53–55]. Considering the differences in higher medical education between China and the West [56], we speculate that the results may be due to the following reasons. In the PBL teaching model, students play a major role in the process of teaching, and teachers facilitate the student learning and support that learning through experimentation, clinical cases, and seminars. The PBL teaching model is rather different from the traditional LBL teaching model in which students often passively accept their teachers’ knowledge. Chinese students have accepted the LBL teaching model for more than 10 years, starting with their primary education, and the PBL teaching model is a novelty that has greatly stimulated students’ interest in learning. [56] Most Chinese medical universities prefer using uniform textbooks for all students, which is quite different from the United States and other Western countries where no uniform textbooks or standard formats of lectures for medical universities exist. [56] In our study, students in the PBL group used the same textbook as students in the LBL group, and they appeared to be better at active learning, which led them to earn more positive examination results. Conversely, the use of the PBL teaching method in Chinese medical higher education is still in its infancy, and the evaluation of the effectiveness of the PBL teaching method is still relatively unsophisticated, particularly for basic medical education. Both teachers and students emphasize course exams [56]; Chinese students’ keen pursuit of positive test scores helps them excel on exams.

The “course type” subgroup analysis shows that the PBL teaching model is more effective when applied in laboratory class settings than in theory-based class settings. Generally speaking, laboratory class exams focus more on execution and analytical skills [23,26,31,36,42,50,53]. The PBL teaching model can inspire students to engage in proactive learning and thinking initiatives, facilitating a stronger grasp of experimental processes and logic. Consequently, students may acquire a deeper understanding of experiments that they conduct, thus enabling them to produce higher quality experimental reports [23]. Therefore, the PBL teaching model would be best applied for laboratory course examinations. On the other hand, while theory courses in several schools also utilize the PBL teaching model, due to limitations on teaching conditions, students are divided into groups but are remain in one classroom, and the number of students in a single classroom can exceed 100 [24,29,37,44,46,49,51]. In contrast, laboratory courses are typically conducted in small groups, which is more suitable for PBL teaching model adoption [23, 26,31,36,42,50,57]. Hence, the advantages of the PBL teaching model relative to the LBL teaching model are more evident when considering laboratory courses.

A number of researchers believe that utilizing a combination of PBL and LBL teaching models for introductory medical courses may improve teaching effectiveness because while the PBL teaching model boosts student initiative and improves proactive learning abilities, the LBL teaching model improves student comprehension of structural knowledge systems and student tendencies to review material after class [44][45]. Our results show that while applying PBL and LBL teaching models in combination can increase excellence rates and examination scores relative to applications of the LBL teaching model alone, there is no evidence that the former approach is more effective than the latter. A detailed review of the studies examined shows that all 17 courses that adopt both PBL and LBL teaching models are theory courses [21,22,24,25,29,34,37–41,44–48,51]. Due to limitations on teaching conditions, teachers can only apply PBL teaching methods when teaching certain modules, and thus the LBL teaching model is still used for remaining classes. As a result, less than half of the lessons conducted over an entire course apply PBL teaching methods. Of the courses that adopt the PBL teaching model exclusively[23,26–28,30–33,35,36,42–44,49,50], 40% (6/15) are laboratory courses[23,26,31,36,42,50]. As mentioned above, laboratory courses may be more suitable for PBL teaching model adoption. Therefore, applying a combination of PBL and LBL teaching models did not result in superior teaching effectiveness relative to the exclusive application of PBL teaching methods.

### Study limitations

Because our study only focuses on undergraduate Chinese medicine higher education, the conclusions may be most applicable to circumstances in China and Asia. Furthermore, this study only evaluated test results—objective outcomes—and it did not assess student attitudes about the PBL and LBL models because these subjective outcomes were not “objectively” measured in the original studies. We also decided to omit a description of subjective outcomes and include only objective outcomes.

The overall quality of the included studies was not high. The mean modified Jadad score for the included studies was only 3.6, and 54.8% of the studies showed modified Jadad scores of less than 4. This study on the effectiveness of the PBL teaching model was also not completely randomized or conducted through double-blind trials. Among the 10 studies on RCT, only two described processes used for randomization sequence generation. These issues may have resulted in low modified Jadad scores and information bias. Higher quality studies on RCT must be examined to better assess the effect of PBL teaching methods.

The three indicators analyzed in this meta-analysis all exhibited marked degrees of heterogeneity. The high heterogeneity may be attributable to the variations in PBL implementation procedures, varying degrees of difficulty in examinations and varying levels of teaching quality among the included studies. Although meta-regression and subgroup meta-analyses were performed, much of the heterogeneity in the subgroup was not eliminated. Heterogeneity among PBL methods is a challenge inherent of all PBL research [13]. Understandings of PBL differ considerably between researchers [58] (e.g., PBL and LBL teaching model teaching hours, course examination methods, etc.). Unfortunately, most of the studies examined did not include detailed information on these factors. Therefore, a random-effect model was applied as the meta-regression model in this study. The heterogeneity maybe affected the reliability of the conclusions of this meta-analysis to some extent.

This study also presents a slight degree of publication bias. Though we conducted a search for literature through the PubMed and EMBASE databases, no non-Chinese studies listed on these databases meet the inclusion criteria. We did not perform a grey literature search, which may have generated information on publication bias. The fixed-effect and random-effect model analysis results of the sensitivity analysis are consistent. This indicates that the analysis results of this research are robust and reliable to a certain degree.

## Conclusions

PBL teaching model application in introductory undergraduate medical courses can increase course examination excellence rates and scores in Chinese medical education system,. The PBL teaching model is more effective when applied in laboratory course settings than when applied in theory-based course settings.
